# Cell alignment on novel polymeric micro-hollow fiber membranes for neural and musculoskeletal tissue engineering

**DOI:** 10.1007/s00441-026-04075-4

**Published:** 2026-05-23

**Authors:** Scott J. Allan, David R. Jenkins, Anna Osborne, Rachael Wood, Georgios Mikalef, Cinzia Amieni, Luca Adly Megalaa Shokralla, Zoe Schofield, Ivan Wall, Eric Hill, Marianne J. Ellis, Patricia Perez Esteban

**Affiliations:** 1 Department of Chemical Engineering, University of Bath, Claverton Down, Bath, BA2 7AY UK; 2 College of Health and Life Sciences, Aston University, Aston Triangle, Birmingham, B4 7ET UK; 3 Department of Chemical and Process Engineering, University of Canterbury, Christchurch, New Zealand; 4 Institute of Translational Medicine, University of Birmingham, Heritage Building, Mindelsohn Way, Birmingham, B15 2TH UK; 5 Institute of Immunology and Immunotherapy, University of Birmingham, Birmingham, B15 2TT UK; 6 Department of Chemistry, Loughborough University, Loughborough, LE11 3TU UK; 7 Aston Institute for Membrane Excellence, School of Biosciences, College of Health and Life Sciences, Aston University, Birmingham, UK

**Keywords:** Cell alignment, Contact guidance, Neural tissue engineering, Skeletal tissue engineering, Micro-hollow fiber membranes, Single orifice-spinning, Cultured meat

## Abstract

Precise positioning and alignment of specific cell types, such as those in the central nervous system and the muscular system, is essential for their functional integration, their migration, and proliferation in vivo. Cell alignment in physiologically relevant tissue models and constructs is challenging to reproduce in vitro unless a three-dimensional scaffold is used. This study demonstrates that cell alignment can be guided quickly, inexpensively, and efficiently using polymeric micro-hollow fiber membranes. These micro-hollow fiber membranes are fabricated via single orifice wet spinning from biocompatible polymers—polystyrene and polycaprolactone. The physicochemical characterization of the micro-hollow fiber membranes confirmed their unique architecture, presenting a special patterning on their outer surface. To establish their potential as a platform for cell alignment via contact guidance, the viability, and degree of alignment of relevant cell lines were evaluated when cultured on the micro-hollow fiber membranes. NG108-15, olfactory ensheathing cells and SH-SY5Y cells were used with the aim to simulate the microspatial distribution of cells within the spinal cord, and C2C12 myoblasts were selected to mimic the highly organized structure seen in muscle tissue. Moreover, differentiation of SH-SY5Y cells was successfully induced while cells remained aligned with respect to the micro-HFM’s axis. The degree of alignment in all cases was quantified via image analysis in combination with the Fast Fourier Transform algorithm method. This work establishes a platform with very particular micro-topographical features that can be employed to direct growth, orientation, and even differentiation of various cell types for tissue engineering and in vitro modelling.

## Introduction

Tissue engineering for repair, regeneration and recreation of physiologically representative and functional tissues involves integrating physical scaffolds to guide cell growth, particularly when cell orientation is essential. In the case of the central nervous system (e.g., spinal cord), the aim of these scaffolds is to enable signal conduction, while retaining cells in a parallel configuration (Alizadeh et al. 2019). For instance, spinal cord motor neurons are the longest cell type in the human body. Encouraging their growth to extreme lengths whilst maintaining their morphology in vitro is a challenge (Suzuki et al. 2023; Stifani 2014). Cellular scaffolds may be used to generate highly organized structures that resemble the in vivo environment (Lizarraga-Valderrama et al. 2019; Miller et al. 2002; Rajnicek et al. 1997).

Another example where cell localization and directionality play a key role is skeletal muscle (Kim et al. 2024; Shimizu et al. 2009). Tissue engineering in this area is applicable to numerous fields and functions, including regenerative medicine (transplantable grafts and cell therapies), drug screening and toxicology, bioactuators, and more recently for consumption as cultured meat (Santos et al. 2023; Ostrovidov et al. 2014; Vandenburgh 2010). To engineer the structure and anisotropy of skeletal muscle as seen in vivo requires the uniaxial organization and parallel alignment of muscle fibers. This is necessary to produce muscle tissue that mimics the morphology of natural skeletal muscle, and for cultured meat, the texture will be a result of many factors, inclusive of muscle fiber maturation and alignment (Ben-Arye & Levenberg 2019). Pre-alignment of cells prior to muscle fiber formation has been shown to increase fiber formation and ensure their alignment (Altomare et al. 2010; Lam et al. 2009).

Methods for achieving cell alignment in vitro include mechanical stimulation such as uniaxial strain and stretching of substrates, electrical stimulation, and the manipulation of the mechanical properties of the substrate, such as stiffness and surface morphology through the introduction of specific topographic features to support contact guidance (Tamiello et al. 2016; Hosseini et al. 2012; Bettinger et al. 2009; Engler et al. 2004; Wilkinson et al. 2002; Vandenburgh & Karlisch 1989). Contact guidance from surface topography has been shown to be a result of both the geometry and size of surface features, with parallel grooves the most widely used feature to align muscle cells in a uniform direction (Ostrovidov et al. 2014; Hume et al. 2012).

The application of hollow fiber membrane technology is very well established in the field of tissue engineering (Eghbali et al. 2016). Hollow fiber membranes (HFMs) constitute, among other kinds of three-dimensional (3D) constructs, the scaffolds required for cell proliferation and differentiation both as a platform for scaled-up expansion and maintenance of culture for the production of viral vectors and recombinant proteins (hollow fiber bioreactors), and as support structures for tissue repair. A 3D culture scaffold where cells are grown around HFMs holds a number of advantages over traditional planar (2D) cultures, since they are able to better represent the in vivo environment and the complex cellular interactions that occur between cells and their surroundings (Wung et al. 2014). The macro-architecture of the hollow fiber membrane provides a highly permeable barrier that can act as a scaffold for adherent cells while maintaining their constant requirements in terms of oxygen and nutrients (De Napoli et al. 2011).

There has been considerable interest in the development of HFMs with very small diameters to be used in the manufacture of more compact membrane modules for higher efficiency separation processes in industry (Yao et al. 2012), but also to explore new applications in areas such as tissue engineering (Tuin et al. 2014). The choice of fabrication method of HFMs with diameters down to the micro-scale determines their properties and therefore their applicability. There are various techniques that can be used to fabricate membranes with dimensions in the micro-scale, such as electrospinning and dry/wet spinning with single or double concentric orifices. Although electrospinning is widely used and an efficient technique for the fabrication of micro- and nanofibers as well as nanotubes, it has some drawbacks in terms of reproducibility and accuracy. This is mainly due to the complex physical processes that occur within the electrified jet, and also environmental parameters such as temperature and humidity (Persano et al. 2013). Furthermore, the presence of additives during electrospinning involves fast solvent evaporation, which makes the porous structure of the HFMs difficult to control and leads to the selection of other methods (such as non-solvent induced phase separation) over electrospinning (Chen et al. 2015). These solvents may also be toxic if the scaffolds are to be used as cell culture platforms (Hong et al. 2019). Single orifice wet spinning, on the other hand, has the potential to reduce the diameter and thickness of the HFMs, and the lack of a bore solution yields a more porous inner wall compared to double orifice spinnerets (Fashandi et al. 2015). Additionally, there are fewer control variables in single orifice wet spinning when compared to coaxial spinning with a concentric orifice—there is only one dimension to be considered (the diameter of the single orifice), and only one flow rate to be optimized as opposed to the flow rates of the inner and outer fluids (i.e., the polymer dope and non-solvent) in concentric orifice spinning. In this work, we explore the production of micro-hollow fiber membranes (micro-HFMs) using a single-orifice spinneret via wet spinning due to its simplicity compared to electrospinning or the conventional concentric orifice spinning process. Avoiding the inner needle in the fabrication process eliminates the challenge of achieving a uniform central lumen, which is particularly important to attain a robust and reproducible process. We selected polystyrene and polycaprolactone as biocompatible polymers for cell culture, and we took advantage of the micro-HFMs’ external topography to grow and align different cell types whose spatial location and directionality is crucial for their physiological function.

## Materials and methods

### Micro-hollow fiber membrane fabrication

**Fig. 1:**
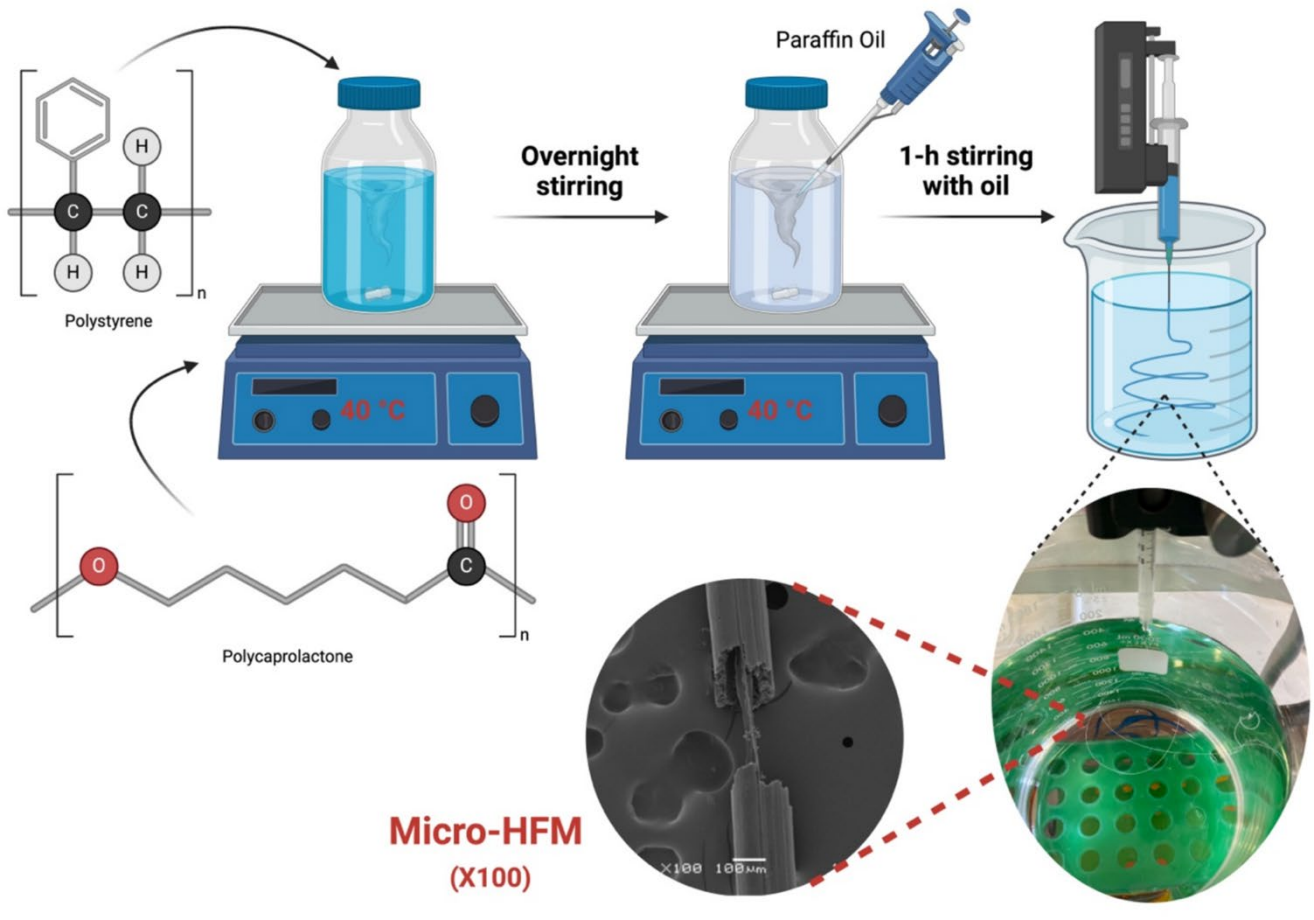
Experimental set-up for micro-HFM fabrication using a single-orifice spinneret. Polystyrene and polycaprolactone were dissolved in NMP overnight before the addition of paraffin oil, which acted as a porogen agent. The polymer/oil mixture was loaded onto a syringe and spun vertically through a blunt needle (27G, 1″ long) into deionized water at a flow rate of 0.01 mL min.^−1^, forming long continuous strings. The resulting micro-HFMs included a hollow-core micro-HFM partially occupied by an internal fiber. Image created using BioRender (www.biorender.com)

A phase inversion, single-orifice spinneret method was followed to fabricate the micro-HFMs, adapted, optimized from the protocol described by Yao et al. (2012) and shown in Fig. 1. The casting solution was prepared in a sealable flask; 0.75 g of polycaprolactone (average M_n_ 80,000, Sigma-Aldrich, Dorset, UK), 0.75 g of polystyrene (average M_n_ 280,000, Sigma-Aldrich, Dorset, UK), followed by 8.5 g of 1-Methyl-2-pyrollidone (NMP, Acros Organics, Fisher Scientific, Loughborough, UK) were added and the flask sealed to avoid solvent evaporation. The solution was stirred continuously under gentle heat (approximately 40 °C) until the polymers were fully dissolved (overnight). Once dissolved, 1 mL of paraffin oil (Sigma-Aldrich, Dorset, UK) was added and mixed on a magnetic stirrer for 1 h. A syringe pump (Cole-Parmer) was set up vertically over a 2-L beaker of deionized water. The casting solution was spun through a syringe blunt needle (ADHERE, 27G, 1″ long) of internal diameter 0.203 mm attached to a syringe. It was ensured that the needle was fully immersed in the water during the whole fabrication process. A flow rate of 0.01 mL min^−1^ was used for the polymer mixture. After spinning, the micro-HFM was transferred into a beaker of fresh deionized water to soak for 24 h in order to fully extract the solvent (NMP). The ratio water: NMP (2 L of water: 8.5 g NMP) as well as the soaking time (24 h) was sufficiently large to remove the solvent that may be trapped within the micro-HFMs structure. Following this, the micro-HFMs were soaked in ethanol at 50 °C for 4 h to extract the paraffin oil, forming the lumen and pores of the micro-HFMs.

### Micro-hollow fiber membrane morphological characterization

Morphological characterization and quantitative analysis of the porosity of the novel micro-HFMs were performed using scanning electron microscopy (SEM). The micro-HFMs were immersed in liquid nitrogen and cut into pieces of approximately 5 mm in length and were sputter-coated with gold (Edwards Sputter Coater 5150B) and analyzed by SEM (JEOL JSM-6480LV) with an acceleration voltage of 10 kV. SEM micrographs of the outer surfaces and the cross-sections of the micro-HFMs were obtained. The internal and external diameters, as well as the porosity were estimated using the free software *ImageJ* (https://imagej.net/ij/) via image analysis. Ten measurements of both the internal and external diameters were taken from three independent batches manually and averaged. In the case of porosity, three representative sections of the SEM micrographs were considered, the threshold was adjusted to differentiate between clear and dark (pores) areas, and the percentage of the area covered by the pores was calculated and averaged for each micrograph.

### Nuclear magnetic resonance (NMR) of micro-hollow fiber membranes

**Fig. 2:**
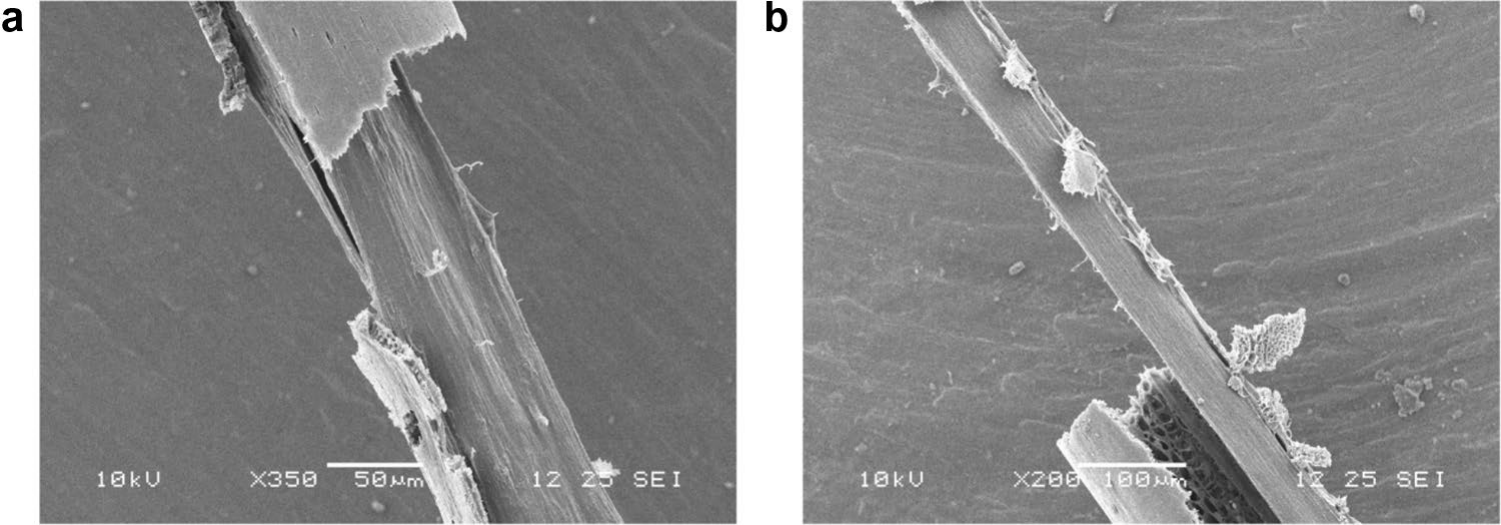
Representative SEM micrographs of micro-HFMs. **a** Separation of the inner structure (× 350), where the inner fiber peels off from the outer encasing micro-hollow fiber membrane; **b** Inner micro-fiber partially separated from the outer encasing micro-hollow fiber membrane, with a visible porous inner wall of the outer micro-fiber (× 200). Scale bars: 50 μm (**a**) and 100 μm (**b**)

NMR spectra of the whole micro-HFMs, and their inner and outer parts individually, were recorded using a Bruker Avance III NMR spectrometer operating at 500.13 MHz for ^1^H. The inner and outer parts were separated manually before analysis in the relevant cases as shown in the SEM micrographs in Fig. 2. Unless otherwise specified samples were analyzed using CDCl_3_ at 25 °C with standard Bruker pulse sequences (Topspin 2.1). ^1^H spectra were acquired with a SW of 20 ppm, and 16 transients. Spectra were referenced using the residual solvent signal, at 7.26 ppm for ^1^H. NMR analysis was provided by the Chemical Characterization and Analysis Facility (CCAF) at the University of Bath.

### Raman spectroscopy of micro-hollow fiber membranes

The chemical composition of the inner and outer parts of micro-HFMs was confirmed via Raman spectroscopy. Samples were prepared by manually pulling micro-HFMs apart, as shown in the SEM micrographs in Fig. 2, and placing them on a glass coverslip. Raman spectroscopy was performed using a WiTec Raman microscope with an excitation source of 515 nm wavelength laser. Spectroscopic analysis was performed across the interface of the internal material and external material (where micro-HFMs had been pulled apart), with a range of 0–4000 cm^−1^. Data was processed using the WiTec results processing software, and acquired images, spectra and diagrams were produced.

### Mechanical characterization of micro-hollow fiber membranes

Mechanical testing of the whole intact micro-HFMs was performed using a Bose ElectroForce® 5500. Micro-HFs were placed vertically in the instrument, and their dimensions were recorded, including the height. A ramp setting was used whereby the micro-HFs were stretched to failure at a constant strain rate of 0.1 mm/s. Measurement parameters were set up accordingly; the preset was − 0.1 g, and the starting level was decided upon the height of the sample. The scan time was 400 s, with 1000 scan points. Cross-sectional area for calculations was determined based on diameter measurements from SEM micrographs. Data from load and displacement was recorded.

### Contact angle measurements

To measure contact angle, a glass microscope slide was dipped in the polystyrene/polycaprolactone/paraffin oil mixture, and immediately submerged in deionized water, simulating the wet-spinning process for micro-HFM fabrication. A high-speed camera (Photron FASTCAM SA3) was used to measure contact angle over 50 frames at 60 frames per second (fps). A 10 µL droplet of deionized water and of RPMI 1640 medium was pipetted on top of the thin layer of polymer mixture and images were taken for three independently prepared samples. The images were analyzed manually using the measuring-angle tool on *ImageJ*. Each angle was measured five times per image left and right and an average was taken to reduce human error.

### Liquid permeation studies

To determine the permeation of water from the lumen of the novel micro-HFMs through their pores, a custom-made glass bioreactor was used. It contained three micro-HFMs of 6 cm in length, and a side port at approximately 2 cm from the inlet that allowed collection of the permeate. Both the inlet and outlet of the glass module were sealed to ensure that the water flowed exclusively through the lumen of the micro-HFMs, and not around them. A constant flow of deionized water was circulated through the lumen of the micro-HFMs at a flow rate of 0.1 mL min^−1^ using a syringe pump (Cole-Parmer). This flow rate was sufficient to allow permeation to occur, giving the possibility to collect quantifiable volumes of permeate, but avoiding damage of the micro-HFMs due to excessive pressure at the module inlet. Samples from the permeate port were taken every 10 min for 2 h, weighed, and the cumulative mass of permeate was calculated.

### Cell culture and differentiation in planar culture

The rat-mouse hybridoma cell lines NG108-15 (Sigma-Aldrich, Dorset, UK) and GFP-expressing SH-SY5Y (kindly gifted by Dr Eric Hill (George et al. 2018)) were used as a model for motor neurons of the spinal cord. NG108-15 cells were maintained in T-75 culture flasks and passaged approximately every 5 days at around 80% confluency. They were maintained in DMEM/F12 (1:1 Dulbecco’s Modified Eagle Medium/Nutrient Mixture F-12) + GlutaMAX™ medium (Gibco™ 2156450) with 10% v/v foetal bovine serum (FBS, Sigma-Aldrich F2442) in a humidified incubator at 37 °C and 5% CO_2_. SH-SY5Y cells were seeded onto tissue culture plastic at a seeding density of 20,000 cells cm^−2^ and maintained in RPMI (Sigma-Aldrich) + 10% FBS (Sigma-Aldrich) + 1% GlutaMAX™ in a humidified incubator at 37 °C and 5% CO_2_. Media was changed every 2–3 days and cells were passaged every 5–7 days at around 80% confluency.

Conditionally immortalized human olfactory ensheathing cells (c-MycER^TAM^-derived hOMCs) were maintained in T-75 culture flasks in a humidified incubator at 37 °C and 5% CO_2_ and passaged approximately every 7 days at around 80% confluency. The hOMCs lines had been previously generated by our team; briefly, they were isolated from olfactory mucosa biopsies and then immortalized using the c-MycER^TAM^ technology (retroviral transduction) (Santiago-Toledo, et al. 2019). To activate cell proliferation, a 1:10,000 dilution of 4-hydroxy-tamoxifen (4-OHT, Sigma-Aldrich H7904) was added to fresh media for media changes and passaging. They were maintained in DMEM/F12 (1:1) + GlutaMAX™ medium (Gibco™ 2156450) with 10% v/v FBS (Sigma-Aldrich F2442). Plates and flasks were freshly coated with Poly-L-lysine (Sigma-Aldrich P4707, 100 µg mL^−1^).

The murine myoblast cell line C2C12 (ECACC 91031101) was used to model skeletal muscle cells. This is an established model for skeletal tissue engineering and has been used in previous research on cultured meat as an application (Allan et al. 2021). Cells were maintained in T-75 culture flasks and passaged approximately every 3 days before reaching 80% confluence. Cells were maintained in proliferation medium consisting of high-glucose DMEM (Sigma-Aldrich D5796) supplemented with 10% v/v fetal bovine serum (FBS, Gibco™, Thermo Fisher Scientific 10270106) and 1% v/v penicillin/streptomycin (P/S; Sigma-Aldrich P4333) in a humidified incubator at 37 °C and 5% CO_2_.

SH-SY5Y cells were differentiated on tissue culture plastic or glass coverslips coated with 200 µL cm^−2^ of 20 µg mL^−1^ Poly-L-Ornithine (pORN) in deionized water, incubated for 4 h, and washed twice with sterile deionized water. Laminin was dissolved in deionized water at a concentration of 10 µg mL^−1^ and incubated overnight. The laminin solution was then removed and washed with DPBS without calcium and magnesium prior to cell seeding. The cell seeding density was 20,000 cells cm^−2^ in RPMI + 10% FBS + GlutaMAX™. SH-SY5Y cells were allowed to attach for 24 h before media was changed to DMEM/F12 + 10% FBS + GlutaMAX™ with 10 µM EC-23 Retinoic Acid (HelloBio) to begin differentiation. Media was changed every other day for 5 days before media was changed to DMEM/F12 + GlutaMAX™ + 50 ng/mL BDNF (Stem Cell Technologies, Germany). This media was replaced every other day until the end of the experiment.

### Cell culture and differentiation on micro-hollow fiber membranes

Micro-HFMs were first plasma treated to improve their wettability and hydrophilicity by exposure to oxygen plasma under a vacuum in a plasma chamber (Diener Zepto) (2 min, power = 50%, O_2_ flow rate = 10 mL min^−1^). Plasma-treated micro-HFMs were then cut to 1-cm-long fibers and placed in a well of an ultra-low attachment 24-well plate (Fisher, 10327701). Micro-HFMs were sterilized by soaking in 70% ethanol for 1 h, washed once with the corresponding growth media and then pretreated with growth media for 3 h. The outer surface of micro-HFMs was seeded with the cell types described previously, at a seeding density of 20,000 cells cm^−2^ (relative to the surface area of the well). In the case of SH-SY5Y cells, micro-HFMs were coated prior to seeding as shown above.

### Cell staining and imaging

Live/Dead staining of cells was used as a qualitative measure of scaffold cytocompatibility, and a calcein-AM/ethidium homodimer (EH) staining kit (Fisher Scientific, 12353643) was selected. The working solution was made up as follows: 1 mL calcein-AM was transferred to 1 µL EH and mixed with PBS at a 1:1 dilution. This stain was used for micro-HFMs in a 24-well plate; 150 µL of staining solution was added to each well, rocked gently for 15 min at room temperature and imaged using either a Leica inverted microscope (Leica DMI4000B) or an EVOS M5000 microscope 3 and 6 days after seeding. SH-SY5Y differentiation was observed via immunofluorescent staining of βIII tubulin (Invitrogen), 1:500 dilution, with Donkey Anti-Mouse Rhodamine (red) secondary antibody (Jackson ImmunoResearch, 112581). Cells were then imaged at specific time points, with the addition of mounting medium containing DAPI (Fluoroshield). Live/Dead assays were used to quantify % viability. Three independent experiments were performed, and five images were analyzed per repeat using *ImageJ*. Briefly, images were converted to 8-bit, the threshold was adjusted manually and the number of cells in green (Live) and red (Dead) were counted. Cell viability is shown as % number of Live cells with respect to the total number of cells counted in each image.

### Directionality analysis

Cell alignment was quantified using directionality analysis. Briefly, five images were obtained per condition per biological repeat after Live/Dead staining. Images were modified in Adobe Photoshop® using shake reduction to sharpen the images. Images were rotated until the micro-HFM was at 180° across the screen. This ensured that the peak that occurred due to the micro-HFM would be at 0° and therefore would not impact the results. Images were cropped to remove any white spaces on the edge of the image that were present after rotation. *ImageJ* was used to convert the images to 8bit and normalize the contrast (set to 0%) to account for different brightness settings of the images. Once all the images were normalized, they were processed using *ImageJ*’s “directionality” plugin using the Fast Fourier Transform (FFT) algorithm method (Fourier components methods, within the “directionality” plugin) (Liu 1991). FFTs are widely used for image analysis of polymer systems and have been shown to be valid for differentiating polymer structures and alignment (Zhu et al. 2018; Deravi et al. 2017). Directional filters were used in the Fourier domain to process images composed of linear patterns, such as cell alignment along fibers as used in this research (Liu 1991). The plugin split the image into pixels and calculated the Fourier power spectra of the pixel, which generated the spatial frequencies of cells/fibers present between 0 and 180° (Deravi et al. 2017). These values were analyzed using polar coordinates and the power was measured for each angle using spatial filters (Zhu et al. 2018). These raw data could be extracted from the program for comparative analysis.

### Statistical analysis and data accessibility

All the results are presented as the average value from three (*N* = 3) independent experiments unless stated otherwise, and the error bars represent the standard error of the mean (SEM) (unless stated otherwise). One-way ANOVA (95% confidence with Tukey’s post-test) was used to assess significant differences between independent samples where appropriate. The datasets generated and/or analyzed during the current study are available from the corresponding authors on reasonable request.

## Results and discussion

### Morphology and porosity of micro-hollow fiber membranes

**Fig. 3:**
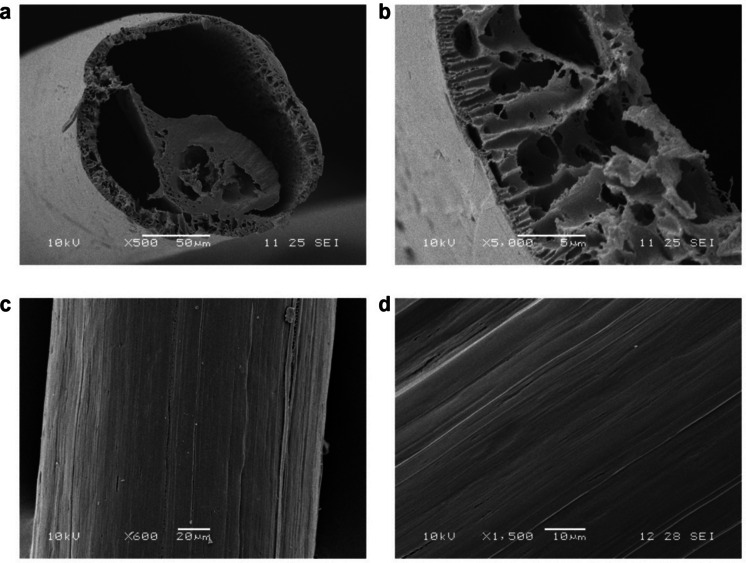
Representative SEM micrographs of micro-HFMs. **a** Cross section (× 500); **b** close-up of the cross section to demonstrate the wall’s porous structure (× 5000); **c** micro-HFM surface, showing the characteristic striations (× 600); D close-up of the micro-HFM’s surface with visible parallel grooves (× 1500). Scale bars: 50 μm (**a**), 5 μm (**b**), 20 μm (**c**), and 10 μm (**d**)

The micro-HFMs’ cross-section at a magnification of × 500 can be seen in Fig. 3a, and the micro-HFMs’ wall at a magnification of × 5000 is shown in Fig. 3b. The most remarkable feature of these novel micro-HFMs is clearly noticeable in Fig. 3a; there is an inner structure present in the center of the micro-HFMs that resembles a hollow fiber itself, and that is detachable manually (Fig. 2). The process of separating the inner part from the outer fiber is clearly shown in Fig. 2. Figure 3c and d show the inherent grooves that result from the fabrication process of the micro-HFMs on their outer surface at a magnification of × 600 and × 1500 respectively.

Despite the presence of the inner “pseudo-hollow micro-fiber,” the lumen is open enough to allow liquid/gas flow through as demonstrated later on in Fig. 8. Image analysis using *ImageJ* revealed that the inner diameter of the micro-HFMs was 144 ± 15 μm (*N* = 10), whereas the outer diameter was 170 ± 11 μm (*N* = 10), giving a wall thickness of approximately 10–13 μm. The image analysis indicated consistent results for completely independently fabricated batches of micro-HFMs, validating the robustness of the process regarding size, which is mainly determined by the needle diameter, in this case 203 μm. As far as the inner structure, or “pseudo-fiber,” is concerned, its diameter was found to be 81 ± 10 μm (*N* = 10), half of the inner diameter of the outer fiber.

A thin but mechanically strong wall is desired, since it facilitates the permeation of compounds in or out of the lumen due to shorter diffusion distances (Taskin et al. 2010). Figure 3b shows a highly porous and thin wall with a sponge-like structure and an estimated porosity of 82 ± 3% (calculated as the area occupied by pores divided by the total area via image analysis using *ImageJ*, *N* = 10). The micro-HFMs presented pores on their outer surface (as seen in Fig. 3b), but also a highly porous inner wall, which would not usually be seen in conventionally spun fibers, since the inner wall is in contact with the non-solvent bore fluid, yielding a less porous wall (Chen et al. 2015). The porous structure generated by extraction of the paraffin oil after spinning is asymmetric, with some narrowing of pores towards the outer surface. The outer surface is smooth with randomly distributed pores. Figure 3a also shows that the cross section is not regular with respect to the thickness of the walls, due to slight variations in the spinning process. This irregularity does not pose an issue in our studies with regards to nutrient distribution for cell culture since the scaffolds were submerged in media and cells were grown on the outer surface of the micro-HFMs. However, this may be considered if the micro-HFMs were incorporated into hollow fiber bioreactors. The irregular cross section would affect the permeation of nutrients from the lumen to the cells on the outside space. However, as the micro-HFMs have an outer diameter smaller than 200 μm and the oxygen diffusion limit is approximately 100–200 μm, at most the diffusion path from the center of the lumen to the cells would be c.a. 100 μm without considering fiber porosity and tortuosity.

Including additives, such as paraffin oil, in the polymer dope during wet spinning usually results in inferior mechanical strength of the micro-HFMs, even though a spongy membrane structure is achieved (Puppi et al. 2012). However, this organization of the pores is preferred over finger-like macro-voids since it improves pore inter-connectivity (Fashandi et al. 2015). Here, we have overcome the issue of mechanical brittleness while maintaining a suitable porosity by introducing a polymer mixture—the polycaprolactone in the formulation of the dope confers flexibility to the resulting micro-fiber as previously shown in the literature (Azimi et al. 2014).

### Structural analysis via NMR and Raman spectroscopy

**Fig. 4:**
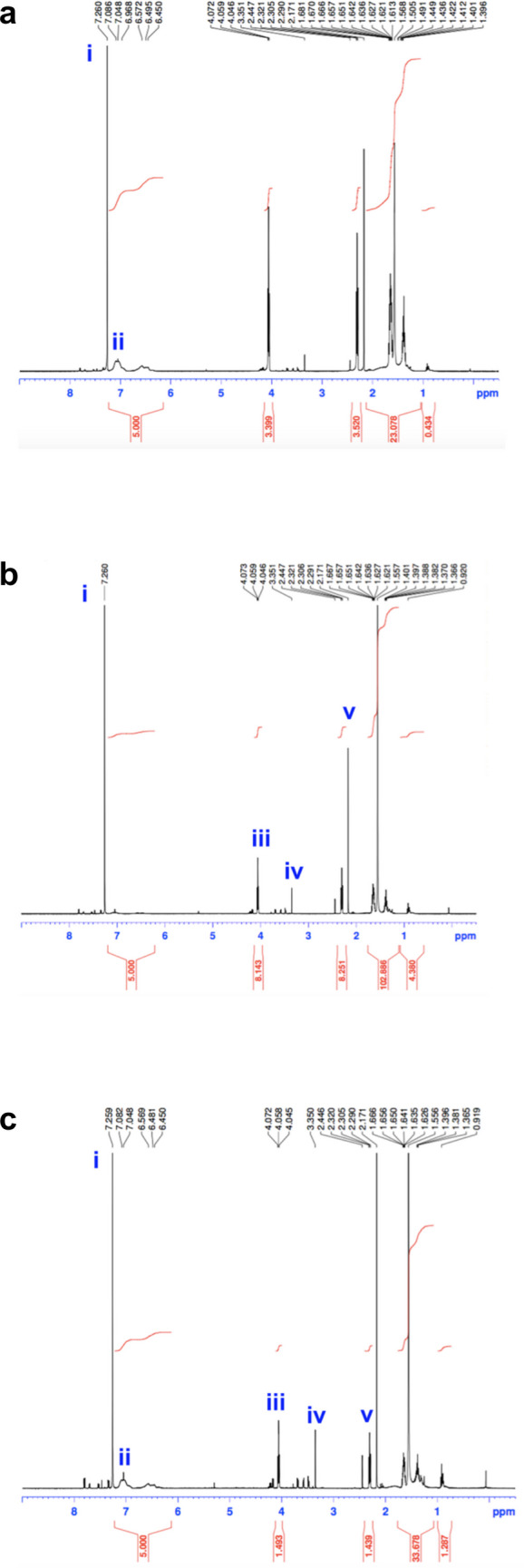
1H NMR spectrum of micro-HFMs. **a** Whole fiber; **b** inner fiber; **c** outer fiber. Chemical shift of the peaks in the spectrum (ppm) is indicated at the top of each spectrum. The integration of relevant peaks is shown in red, and the approximate location in the spectrum of relevant peaks is annotated in Roman numerals in blue

The presence of two completely differentiated structures in the micro-HFMs shown in Fig. 3 introduced the question of the polymer distribution within the double hollow fibers. The polystyrene and polycaprolactone used in this study have different properties in terms of their molecular weights (M_w_ 280,000 and 80,000, respectively), and viscosities when dissolved at the same concentration in NMP (0.273 ± 0.008 Pa s and 3.543 ± 0.464 Pa s at 25 °C respectively) (determined experimentally via a High-Resolution Torque Rebalance C-VOR rheometer (Bohlin Instruments), data not shown) and most importantly, their solubility in NMP. While polystyrene dissolves well in NMP, polycaprolactone does not fully dissolve at room temperature in NMP. It was suspected that partial separation of the polymers occurred during the fabrication process of the micro-HFMs. This hypothesis was supported by the observed different mechanical properties of the inner and outer micro-fibers; elastic and strong internally, and delicate and brittle externally, resembling the properties of hollow fibers fabricated with polycaprolactone and polystyrene alone respectively (data not shown). The hypothesis was confirmed using ^1^H-NMR analysis as can be seen in Fig. 4.

All spectra shown in Fig. 4 present a peak at 7.26 ppm (peak i), which corresponds to the residual solvent signal (CDCl_3_). There is a peak at circa 7 ppm (peak ii) in the outer fiber spectrum (Fig. 4c), which can be assigned to the aromatic hydrogens present in the polystyrene chemical structure (Bovey et al. 1965). This peak cannot be observed in Fig. 4b (inner fiber), indicating a much smaller proportion of polystyrene in the inner core, as suspected. Both the inner and outer fiber spectra (Fig. 4b and C respectively), show peaks in the area of 4 ppm and 3.3 ppm (peaks iii and iv respectively), which are characteristic for polycaprolactone (Ramírez Hernández et al. 2013; Kerman et al. 2005). Additionally, there is a larger peak in the inner fiber spectrum (Fig. 4b) in the region of 2.5–2.2 ppm (peak v), when compared to Fig. 4c. This further demonstrates a larger proportion of polycaprolactone in the inner fiber (approximately 8 times larger). A quaternary membrane-forming system like the one we show here, consisting of a nonsolvent (water), a solvent (NMP), and two membrane forming polymers is thermodynamically very complicated. Our polymers mix well, as indicated by their similar Hildebrand solubility parameters (18.61 MPa^1/2^ for polystyrene (Welker 2012) and 20.57 MPa^1/2^ for polycaprolactone (Madsen et al. 2015)). This means that they can be considered as an intertwined network of polymer molecules “swollen” with solvent and nonsolvent molecules. Initially in the process of phase inversion, the movement between the two polymers is negligible compared to the movement of the water and NMP (Boom et al. 1994), and water penetrates through the polymer mixture. However, in the longer time scale, the movement between the two polymers occurs again—they tend to separate for entropic reasons: two polymers have very low entropy of mixing (Boom et al. 1994). This explains the presence of the “pseudo-fiber” in the middle, where polycaprolactone moves away from polystyrene for thermodynamically favorable reasons. This is relevant and effectively very useful for tissue engineering applications to achieve a specific spatial distribution of two or more different cell types; for example, to create artificial blood vessels or simulate the blood–brain barrier.

**Fig. 5:**
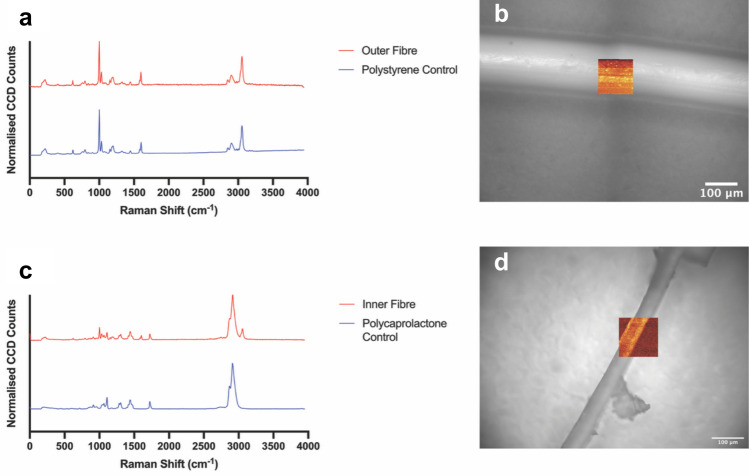
Raman spectroscopy of micro-HFMs. **a** Raman spectra of the outer fiber (red) compared with a polystyrene control (blue). Spectra are plotted as normalized CCD counts (to the highest peak) versus Raman shift (cm^−1^), highlighting characteristic vibrational peaks for polystyrene at 1001 cm^−1^ (ring breathing mode) and 1602 cm^−1^ (ring-skeletal stretch). **b** Microscopic image of the outer fiber with an overlaid Raman heat map showing the area scanned for the Raman spectrum of the outer fiber in **a**. Scale bar: 100 µm. **c** Raman spectra of the inner fiber (red) compared with a polycaprolactone control (blue), showing distinct vibrational features at 1110 cm^−1^ (skeletal stretching) and ~ 1300 cm^−1^ (CH_2_ vibrations of methylene groups). **d** Microscopic image of the inner fiber with an overlaid Raman heat map illustrating the area scanned for the Raman spectrum of the inner fiber in **c**. Scale bar: 100 µm

The distribution of polymers in the distinct inner and outer fibers was further confirmed via Raman spectroscopy as shown in Fig. 5.

Figure 5 illustrates the use of Raman spectroscopy to confirm the chemical identity and spatial distribution of polymers within the micro-HFMs and support the NMR results shown in Fig. 4.

Figure 5a shows the Raman spectra of the outer fiber (red) compared with a polystyrene control (blue), which was obtained from the exact same material used for the fabrication of micro-HFMs (polystyrene, average M_n_ 280,000, Sigma-Aldrich, Dorset, UK). There are clear peaks at 1001 cm⁻^1^ and 1602 cm⁻^1^ corresponding to the ring breathing and ring-skeletal stretch modes of polystyrene (Mayorga et al. 2025). These spectral features are consistent with established vibrational signatures of polystyrene. The Raman heat map in Fig. 5b confirms the spatial localization of these signals across the scanned region, indicating a homogeneous distribution of polystyrene on the fiber surface.

Figure 5c presents the Raman spectra of the inner fiber (red) compared with a polycaprolactone (PCL) control (blue) (polycaprolactone, average Mn 80,000, Sigma-Aldrich, Dorset, UK), showing characteristic peaks at 1110 cm⁻^1^ (skeletal stretching) and ~ 1300 cm⁻^1^ (CH₂ vibrations). These features are well-documented for polycaprolactone and confirm its presence in the inner fiber (Baranowska-Korczyc et al. 2016). The corresponding Raman heat map in Fig. 5d supports the spatial confinement of polycaprolactone within the fiber core.

The ability to spatially resolve polymer domains using Raman spectroscopy is particularly valuable in multi-material scaffolds such as the micro-HFMs presented here, where compositional gradients or interfaces can influence mechanical and biological performance.

In the context of hollow fiber membranes, Raman spectroscopy offers a robust approach for verifying polymer identity and uniformity, which is essential for reproducibility and performance in cell culture and tissue engineering (Sharikova et al. 2020). The integration of Raman mapping with microscopy, as shown in Fig. 5, provides a powerful platform for scaffold validation, ensuring that the spatial arrangement of materials aligns their intended use.

### Mechanical characterization

**Fig. 6:**
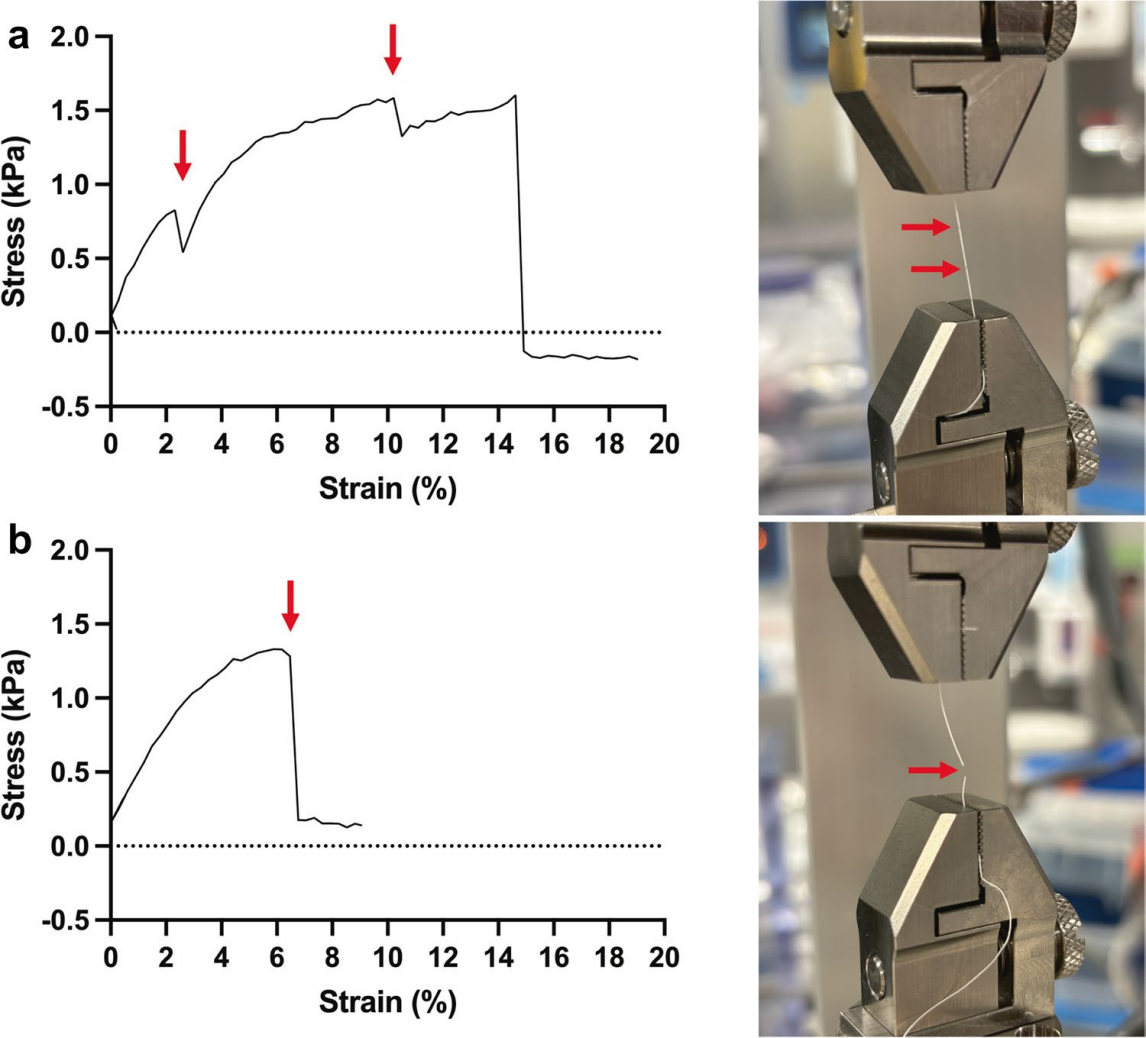
Mechanical characterization of micro-HFMs under maximum uniaxial tensile stress. **a** Representative stress–strain curve for polystyrene/polycaprolactone micro-HFMs. There are three main mechanical transitions, with the first two marked by red arrows: an initial yield point at approximately 2% strain, a secondary yield point at ~ 10% strain, and failure at ~ 15% strain (final break). **b** Representative stress–strain curve for polystyrene only micro-HFMs, highlighting a yield point at ~ 6% strain followed by rapid failure. The corresponding images display samples in the tensile testing setup, with red arrows marking the mechanical transitions

Figure 6 presents the mechanical characterization of micro-HFMs under uniaxial tensile stress, comparing composite polystyrene/polycaprolactone fibers with polystyrene-only fibers. The stress–strain curves and corresponding tensile testing images provide insight into the mechanical behavior and failure mechanisms of these materials.

Figure 6a shows the stress–strain response of the polystyrene/polycaprolactone micro-HFMs, revealing three distinct mechanical transitions. The initial yield point at approximately 2% strain likely corresponds to the onset of plastic deformation in the polycaprolactone phase, which is known for its ductility and low modulus. The elongation at this point was measured as 2 mm. The secondary yield point at ~ 10% strain may indicate the engagement of the polystyrene phase, which contributes to increased stiffness and resistance to further deformation, producing a further 1.5 mm elongation. Final failure occurs at ~ 15% strain, suggesting that the composite structure allows for greater elongation and energy absorption before rupture. The corresponding image confirms these transitions, with visible deformation and fiber elongation marked by red arrows. The Young’s modulus was 0.35 kPa, calculated as the slope at the initial linear region, and the ultimate tensile strength was 1.60 kPa.

In contrast, Fig. 6b displays the stress–strain curve for polystyrene-only micro-HFMs as a control, which exhibit a single yield point at ~ 6% strain followed by rapid failure. This behavior is characteristic of brittle polymers like polystyrene, which lack the capacity for significant plastic deformation. There is therefore no elongation at tear. The Young’s modulus was 0.33 kPa, calculated as the slope at the initial linear region, and the ultimate tensile strength was seen as 1.33 kPa. The absence of a secondary yield point and the steep drop in stress post-yield indicate limited toughness and poor strain accommodation. The tensile testing image supports this interpretation, showing abrupt failure with minimal elongation.

These results highlight the mechanical advantages of incorporating polycaprolactone into the fiber architecture. The composite micro-HFMs demonstrate improved ductility, multi-phase deformation behavior, and delayed failure compared to polystyrene-only fibers. This is consistent with previous studies showing that blending or layering polymers with complementary mechanical properties can enhance scaffold performance in tissue engineering applications (Echeverria Molina et al. 2021).

Moreover, the presence of distinct mechanical transitions in the composite fibers suggests phase-specific contributions to the overall mechanical profile, which could be tuned to match the requirements of different tissue types. For example, scaffolds intended for load-bearing tissues may benefit from the enhanced toughness and strain tolerance observed in the composite fibers.

### Contact angle and wettability of micro-HFMs

**Fig. 7:**
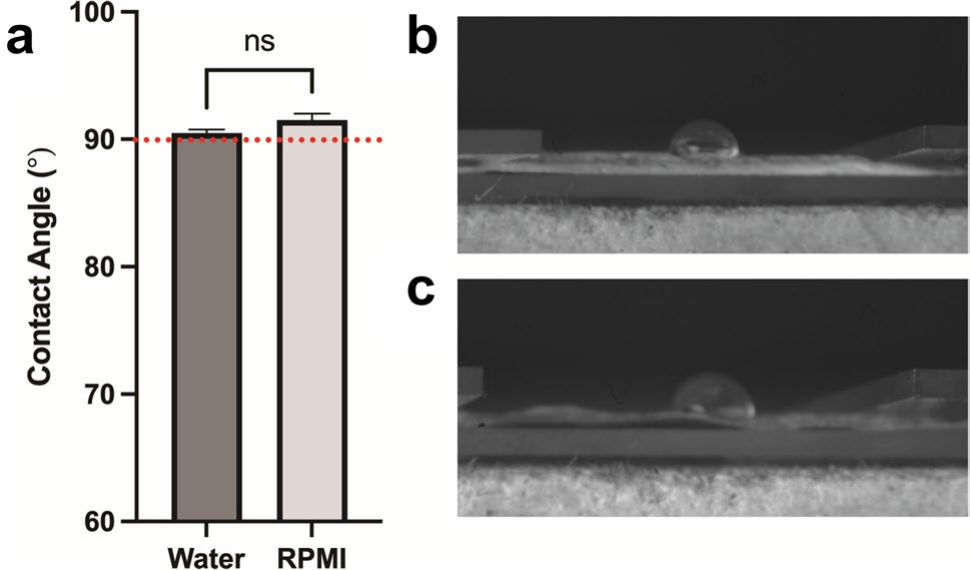
Contact angle as a measure of wettability of polystyrene/polycaprolactone film layers. **a** Quantification of contact angle using water and RPMI 1640 medium as wetting agents. Average values of three independent experiments are shown, for which 5 images were analyzed per independent repeat. Error bars represent SEM and statistical differences were assessed using a *t*-test. **b** Representative image of contact angle using water as the wetting agent. **c** Representative image of contact angle using RPMI 1640 medium as the wetting agent. Contact angle values approximated and slightly exceeded 90° for both cases, indicating relative hydrophobicity of the surfaces

Water and medium (RPMI 1640) contact angle measurements shown in Fig. 7 indicated that the polystyrene/polycaprolactone surface exhibits a distinct wettability profile, reflecting the balance between the hydrophobic polystyrene phase and the more polar polycaprolactone phase. The observed contact angle suggests that the surface chemistry and any phase segregation or roughness effects are governing the interfacial behavior of the sample (Zheng et al. 2006).

Polystyrene is typically more hydrophobic (Li et al. 2007) and the results shown in Fig. 7 corroborate that the polymer distribution in the thin layer of polymer studied resembles that observed in the micro-HFMs: the surface is relatively non-wetting (contact angle close to 90°) suggesting the polystyrene-rich nature of the surface. The poor wettability of polymeric hollow fiber membranes has previously been reported in the literature (Meneghello et al. 2009), highlighting the need for plasma treatment and surface coatings as performed in our study if they are to be used in tissue engineering applications.

### Liquid permeation of micro-HFMs for 2 h

**Fig. 8:**
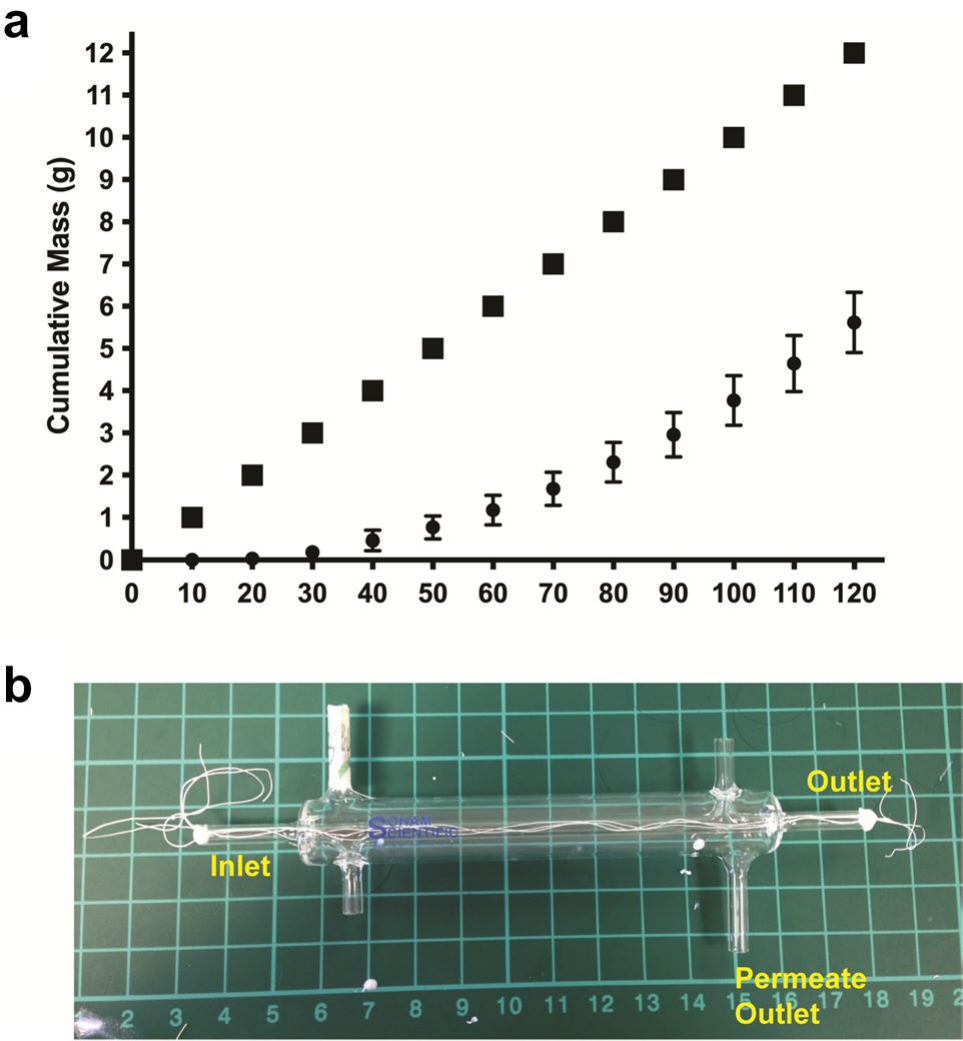
**a** Liquid permeation through the porous walls of micro-HFMs determined as cumulative mass of permeate over time compared to the cumulative inlet mass for a flow rate of 0.1 mL min^−1^ pumped through the inner lumen of micro-HFMs (*N* = 3, Error bars represent the standard deviation of three independent repeats). **b** Experimental set-up for liquid permeation experiments, including the threaded and sealed micro-HFMs in the custom-made glass module

A flow rate of 0.1 mL min^−1^ of deionized water was used, which was distributed through the lumen of the three micro-HFMs. This study was initially conducted for 2 h to observe the initial moments of permeation before reaching steady state. The cumulative mass of permeate that perfused through the porous wall of the micro-HFMs is shown in Fig. 8.

It is clear that permeation occurs through the fiber walls as evidenced by the increase in permeate collected over time in Fig. 8. After 60 min, approximately 6.0 g of water would have been cumulatively run through the lumen of the micro-HFMs in the module. From Fig. 8, it can be calculated that circa 19.6% of that water had permeated through the pores after 60 min, and 46.8% permeation was achieved after 120 min. This indicates the suitability of our constructs for cell culture as media can be perfused through the micro-HFMs, which is a distinctive feature not seen previously in the literature in hollow fibers in the micro-scale for similar applications, where the scaffolds are directly immersed in media (Morelli et al. 2021).

### Cell alignment and differentiation on micro-hollow fibers

Various cell types whose positioning and directionality are key for their function were selected and grown on the micro-HFMs. Previous studies have shown that generally motor neurons are aligned and static in the spinal cord (Kim et al. 2019), and their cell alignment and precise location are essential for axon guidance and migration. Considering the same physiological environment, after a spinal cord injury occurs, a scar tissue is formed. The astrocytes within, which turn hypertrophic following disruption of the blood–brain barrier (Cregg et al. 2014), also become misaligned, leading to changes in the extracellular matrix that surrounds them (Raisman 2001). It is therefore clear that directionality in the central nervous system determines both cellular function and dysfunction, as well as alterations of the physiological microenvironment. NG108-15 cells were chosen as a model cell line here to study neuronal alignment, and olfactory ensheathing cells (OECs) were included due to their capability of inducing axon regeneration (Gómez et al. 2018) and as a surrogate to represent the glial population in the spinal cord (e.g., astrocytes). Additionally, in the emerging field of cellular agriculture, and the production of cultured meat, there remain challenges around reproducing the biology of skeletal in vitro; skeletal muscle cells as a protein alternative need to first proliferate to the necessary scale prior to differentiation to achieve the desired protein content. Beyond this basic form of cultured meat, the production of “full cut” meat requires not only multiple cell types, but cells growing in an organized fashion (Stephens and Ellis 2020; Stephens et al. 2018). Skeletal muscle is characterized by its three-dimensional anisotropic structure that comprises highly aligned muscle cells, hence why a model cell line for myoblasts (C2C12 cells) was selected to demonstrate potential alignment on our micro-HFM system.

**Fig. 9:**
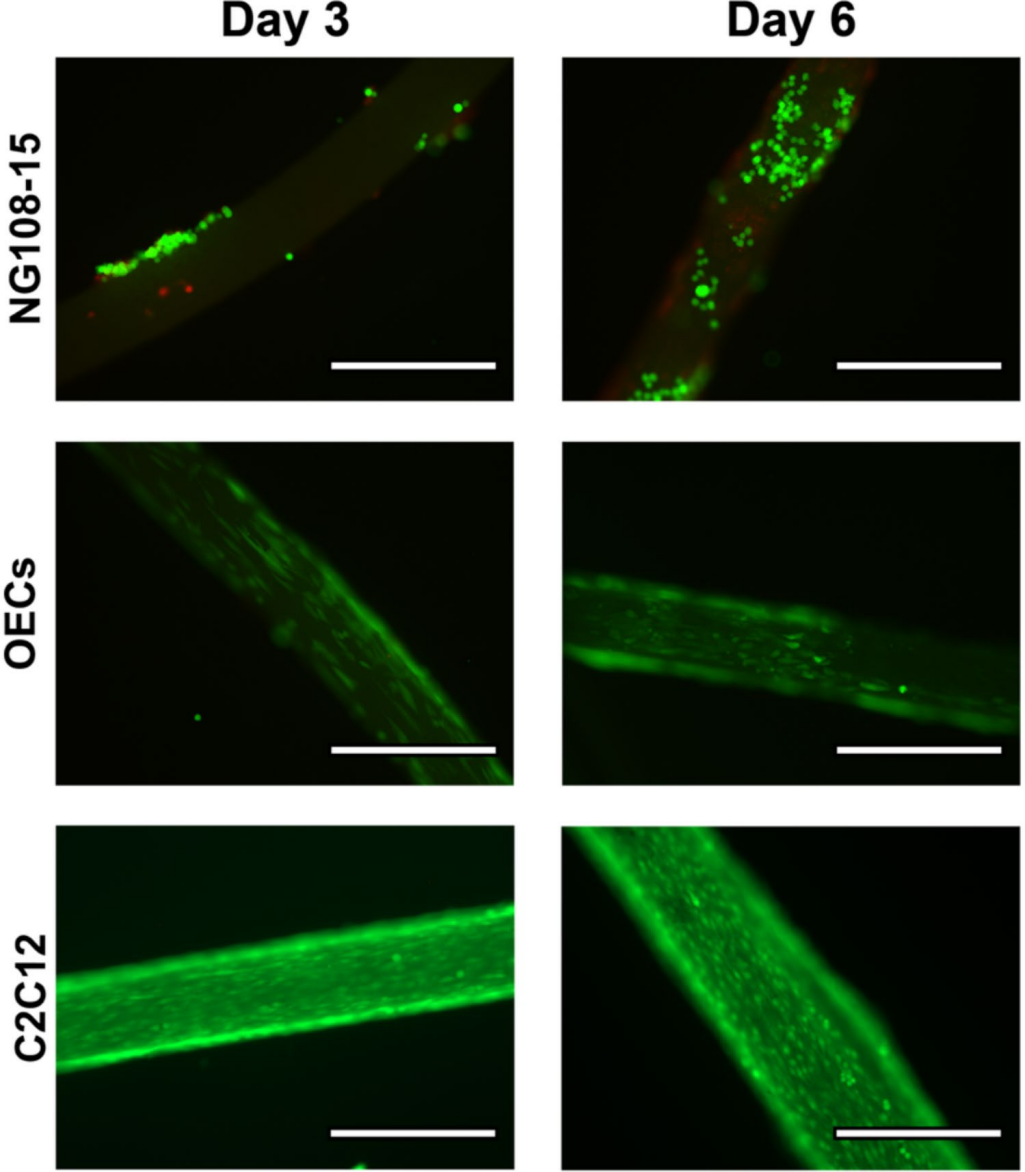
Fluorescent micrographs of Live/Dead-stained NG108-15 s, OECs, and C2C12 Myoblasts (calcein-AM/ethidium homodimer (EH)). All cell types were grown on micro-HFMs for 6 days in three independent biological repeats each. Images were taken at day 3 and day 6. Green cells are live cells (Calcein-AM stain), and red cells are dead cells (EthD-III stain). Scale bars: 400 μm

**Fig. 10:**
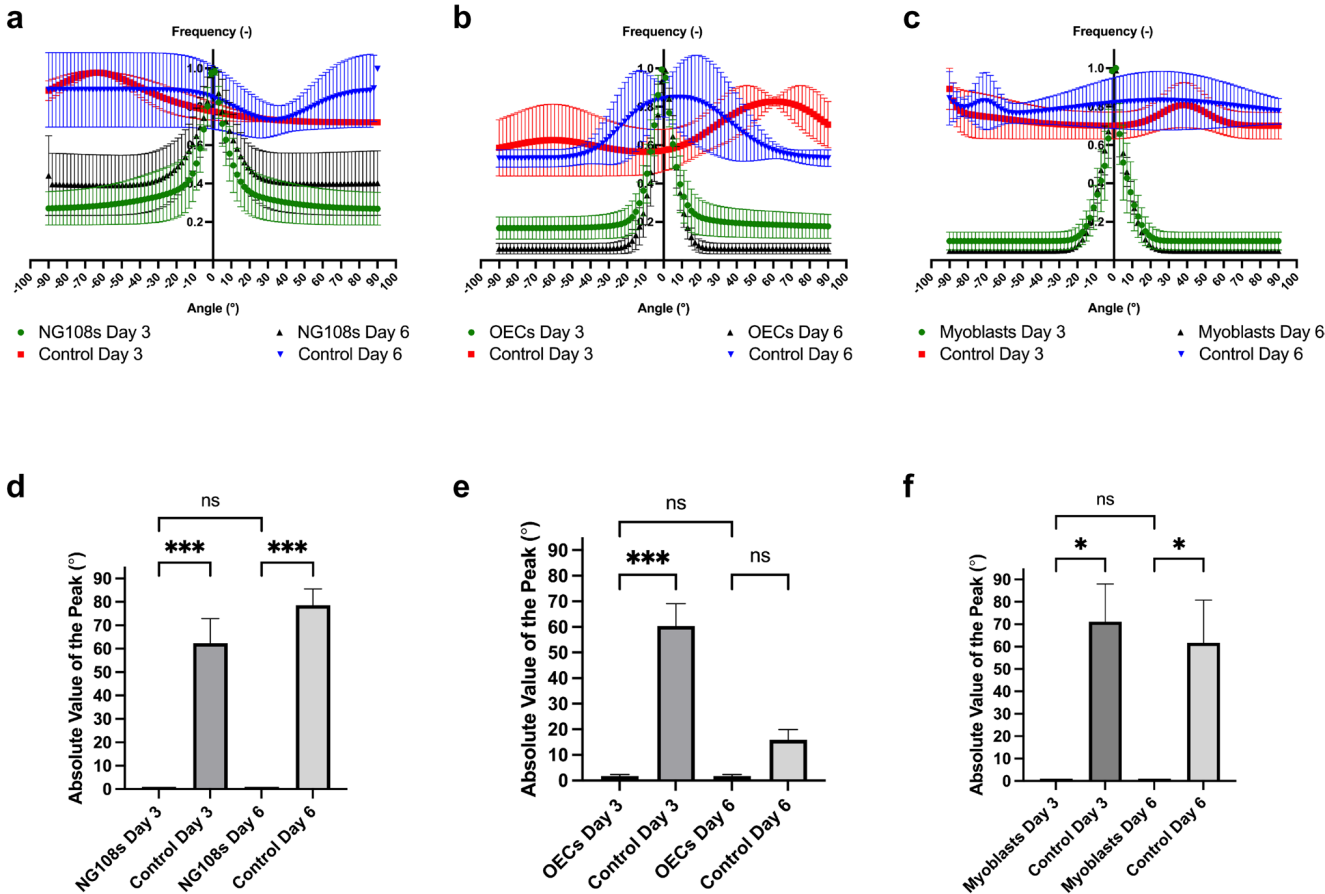
Quantification of cell directionality using the Fast Fourier Transform algorithm method, with cells grown on micro-HFMs on day 3 (green), in planar culture on day 3 (control, red), on micro-HFMs on day 6 (black) and in planar culture on day 6 (control, blue). **a** Directionality of NG108-15 cells. **b** Directionality of OECs. **c** Directionality of C2C12 myoblasts. Data are normalized means to the largest frequency value ± SEM (*N* = 3 independent biological repeats with 15 images analyzed for each repeat). Absolute value of the orientation angle with respect to the micro-HFM axis for the highest frequency directionality peak for **d** NG108-15 cells; **e** OECs; **f** C2C12 Myoblasts. Controls refer to cells grown on 2D planar culture. A value of 0° represents complete alignment. Data are means ± SEM (*N* = 3 independent biological repeats with 15 images analyzed for each repeat). ANOVA with multiple comparisons, 95% confidence, was carried out on the data shown (*P* values: ***P < 0.001; **P = 0.001 to 0.01; *P = 0.01 to 0.05; ns, not significant; *P* ≥ 0.05)

Figure 9 shows that all cell types studied here progressively align to the axis of the outer surface of micro-HFM in the culture period of 6 days. Moreover, they seem to proliferate, as evidenced by the qualitatively higher number of cells that can be seen in all panels in Fig. 9. Scarce dead cells (shown in red) can be seen in some instances, possibly due to attachment issues or lack of cell–cell interactions because of their spatial distribution. The number of dead cells observed is, nevertheless, very small, indicating high cytocompatibility and adhesion to the micro-HFMs (viability 92% ± 5%). In order to quantify the extent of this alignment, image analysis and directionality calculations were performed, based on the Fast Fourier Transform algorithm. This method has previously been used to assess directionality in other fields (Molina et al. 2019). Five images per well, with three wells per biological repeat were analyzed. The results can be seen in Fig. 10 for all cell types, where the frequency, effectively the normalized number of cells (ratio of total cells) that are oriented at a specific angle with respect to the micro-HFM’s axis, is shown. Cells that are completely in line with the micro-HFM’s axis will produce a value of 0 degrees (°), whereas cells in any other orientation will appear at various values of angle in the x-axis. Frequencies were normalized to the highest value in order to be able to compare between cell types (Fig. 10a–c), with the highest number of cells at a specific angle having a frequency of 1.

The controls shown in panels a-c in Fig. 10 represent cell growth on regular planar culture at the same seeding densities (20,000 cells cm^−2^) over the same culture time (6 days). It is evident that cell directionality in two-dimensional planar systems is random (red and blue in Fig. 10a–c); all cells analyzed were oriented at different angles, covering the whole spectrum of orientations. For all cell types grown on micro-HFMs at both time points (day 3, green and day 6, black), there is a very remarkable peak of frequency around 0°, indicating that the majority of the cells are aligned with respect to the micro-HFM’s axis. To further demonstrate the effectiveness of the alignment achieved with these micro-HFMs, we quantified the statistically significant differences between cells grown on planar culture versus those grown on micro-HFMs at day 3 and day 6 by comparing the values of the angles corresponding to the highest frequency (without considering the angle’s sign)—the absolute value of the peak. This is a measure of the most frequent angle of alignment, or in other words, the preferred angle at which cells orient themselves. An absolute value of the peak of 0° represents complete alignment. The results of this quantification are shown in Fig. 10d–f.

As seen in Fig. 10d, the preferred orientation angle for NG108-15 neuroblastoma × glioma hybrid cells grown on micro-HFMs is very close to zero (perfect alignment to the axis), and statistically significantly lower than the orientation angle in two-dimensional culture (control) for both time points (*P* = 0.0006 for day 3 and *P* = 0.0001 for day 6). Some studies have attempted the alignment of NG108-15 s or other neural-like cells on or encapsulated in electrospun fibers (Ranjan et al. 2020; Lizarraga-Valderrama et al. 2019; Quan et al. 2019; Soliman et al. 2018), in multi-channel architectures (Sun et al. 2019), and hydrogel tubes (Muangsanit et al. 2021; Dumont et al. 2019), but never on micro-porous hollow fibers with the advantages of the micro-scale, characteristic surface patterning without the need of specialized equipment, and porosity for mass transfer of oxygen and nutrients (Tuin et al. 2014; Morelli et al. 2021). OEC alignment has been underexamined to date, and the results in Fig. 10e indicate enhanced unidirectionality (OECs versus control at day 3, *P* = 0.0001). This has a notable potential for a combination of tissue engineering and cell therapy-based strategies for the treatment of trauma in the central nervous system (Zhang et al. 2019). Finally, C2C12 myoblasts are statistically significantly more aligned than their counterparts grown on planar culture at both time points (Fig. 10f) (*P* = 0.0194 for day 3 and *P* = 0.0399 for day 6). The alignment of muscle cells here is particularly novel—from a tissue engineering perspective, diameters in the macro-scale normally lead to larger focal adhesions, which promote actin organization and greater cell alignment (Jenkins and Little 2019). Work on larger collagen scaffolds has been conducted for alignment of C2C12 myoblasts, with promising results (Basurto et al 2021). We have achieved the same effect here, but in the micro-scale. Fiber alignment has been previously attempted, focusing only on the physical distribution of several fibers with respect to each other. Here, it is hypothesized that the inherent parallel grooves on the surface of the micro-HFM that result from the fabrication process consequently induce cell alignment and preferential orientation via contact guidance, eliminating the need for further surface patterning.

**Fig. 11:**
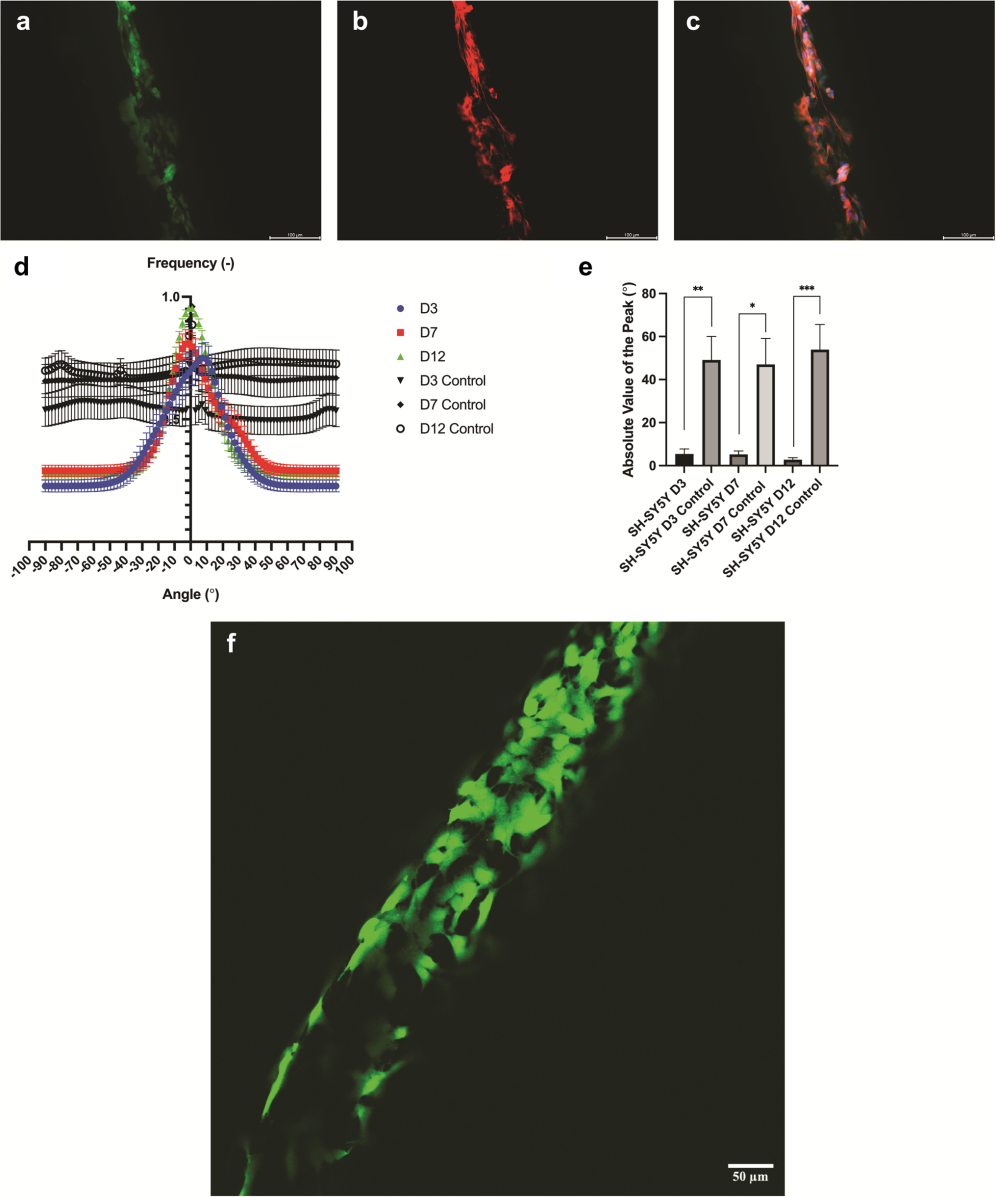
Fluorescent micrographs of aligned SH-SY5Y cells on micro-HFMs. **a** SH-SY5Y cells genetically tagged with GFP; **b** βIII tubulin staining; **c** DAPI merge with **a** and **b**. **d** Quantification of cell directionality using the Fast Fourier Transform algorithm method, with cells grown on micro-HFMs on day 3 (blue), day 7 (red), and day 12 (green). 2D controls for days 3, 7, and 12 are shown in black (triangle, circle, and empty circle respectively). Data are normalized means to the largest frequency value. **e** Absolute value of the orientation angle with respect to the micro-HFM axis for the highest frequency directionality peak for SH-SY5Y cells on micro-HFMs compared to the 2D controls on days 3, 7, and 12. Data are means ± SEM (*N* = 3 independent biological repeats with 15 images analyzed for each repeat). ANOVA with multiple comparisons, 95% confidence, was carried out on the data shown (*P* values: ***P < 0.001; **P = 0.001 to 0.01; *P = 0.01 to 0.05; ns, not significant; *P* ≥ 0.05). Scale bars: 100 μm. **f** Fluorescent micrograph of aligned SH-SY5Y (genetically tagged with GFP) cells on micro-HFMs at × 20 magnification (day 12). Scale bar: 50 μm

Alignment of differentiated GFP-SH-SY5Y cells (green) was seen through fluorescent microscopy as shown in Fig. 11a, stained for βIII tubulin (red) (B), and merged with DAPI (C). These images and Fig. 11f show the cells as well as their neurite extensions growing along the micro-HFMs. This was further quantified through *ImageJ* directionality analysis, using the Fast Fourier Transform (FFT) algorithm.

The 2D control was seen to show no alignment (Fig. 11d), with no distinct peaks for any of the studied timepoints. Quantification of the absolute value of the peak is shown in Fig. 11e in order to directly compare cell directionality over time and to the 2D controls. Figure 11d and e evidence that the degree of alignment of SH-SY5Y increased over time, and more neurites extended with further alignment along the micro-HFMs as seen in Fig. 11c. When compared to the control, there were significant differences at all time points. It has previously been reported that cells can be aligned on 2D substrates through protein patterning, topography and chemical gradients (Chelli et al. 2014; Deumens et al. 2004, Cassanova et al. 2018), but not on a three-dimensional scaffold. These findings underscore the critical role of alignment in promoting cell growth and differentiation, particularly within three-dimensional scaffolds, which offer a more physiologically relevant environment compared to traditional 2D substrates. The ability to guide neurite extension and enhance directional growth on micro-HFMs highlights the potential of aligned 3D architectures to better mimic native tissue organization and improve outcomes in neural tissue engineering (Coyle et al. 2022).

## Conclusions

We have established a quick, simple and cost-effective fabrication method of porous micro-hollow fiber membranes using a novel polymer mixture that generates a three-dimensional construct, which is mechanically robust to allow liquid permeation and has a very particular internal and external architecture. As a proof of concept, we have demonstrated that the external features of these micro-HFMs, i.e., the linear indentations on their surface, promote the self-alignment and directionality for representative cell types of the central nervous system and skeletal muscle. Linear topographies and fibrous/tubular systems have been used for cell alignment previously, but this is the first study to take advantage of both elements together. This work provides a strong basis for the development of functional tissue-engineered models in the fields of disease modelling, tissue regeneration and cellular agriculture, amongst others.

## Data Availability

The datasets generated and/or analyzed during the current study are available from the corresponding authors on reasonable request.
